# Is the Relationship Between Adolescent Social Isolation and Anxiety‐Like Behaviors Altered by Microglia Ablation in Female Long Evans Rats?

**DOI:** 10.1002/brb3.70369

**Published:** 2025-03-09

**Authors:** Matthew A. Blumberg, Ava Shipman, Lidia Olyha, Stephen C. Gironda, Jeffrey L. Weiner

**Affiliations:** ^1^ Department of Translational Neuroscience Wake Forest University School of Medicine Winston‐Salem North Carolina USA

**Keywords:** anxiety, microglia, PLX3397 (Pexidartinib), social isolation

## Abstract

**Objective:**

Despite extensive, cross‐disciplinary research revealing a relationship between early life stress (ELS) and an increased risk for neuropsychiatric disorders, the underlying processes mediating this relationship are not fully understood. Further, the majority of preclinical studies investigating this relationship have not taken sex differences into consideration. A growing body of work suggests that microglia, resident immune cells of the brain, are impacted by ELS and contribute to some of the maladaptive behavioral phenotypes in adulthood. Here, we utilized an adolescent social isolation (aSI) model of ELS in female rats to test the role of microglia in mediating the effects of ELS on anxiety‐related behaviors.

**Methods:**

The present study sought to determine whether microglia ablation during aSI could prevent anxiety‐like behaviors in female Long Evans rats. A colony‐stimulating factor 1 receptor (CSF1‐r) inhibitor, PLX3397, was provided in chow to ablate microglia at the start of the isolation period (postnatal day (P) 21–42). During the aSI period, animals performed a battery of behavioral assays including the open field test, elevated plus maze, and successive alleys test. Following completion of the behavioral assays, brain tissue was collected to confirm the efficacy of PLX3397 and identify changes in microglia population density.

**Results:**

Relative to group‐housed (GH) controls, aSI rats showed increased locomotor activity in the open field test and higher closed‐arm entries on the elevated plus maze. Although PLX3397 effectively ablated microglia across all animals, this treatment had minimal effects on observed aSI‐associated phenotypes.

**Conclusions:**

Together, these data suggest that microglia are not required for behavioral adaptations promoted by aSI. Future studies will be needed to assess the role of microglia in the relationship between ELS and maladaptive behavioral phenotypes.

## Graphical abstract

The present study sought to determine the effects of microglia ablation via a PLX3397 diet during adolescent social isolation (aSI) could prevent anxiety‐like behaviors in female Long Evans rats. Our study demonstrates microglia ablation does not alter behavior on the open field test (OFT), elevated plus maze (EPM), and successive alleys test (SAT).

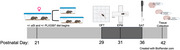

## 1 Introduction

Early life stress (ELS) is recognized as a major risk factor for many mental health disorders, including generalized anxiety disorder (GAD) (Kessler et al. 2010; Lähdepuro et al. 2019; Juruena et al. 2020). The American Psychological Association recently concluded that over one in three Americans report having a diagnosed mental health disorder, with over 19% of those individuals affected by an anxiety disorder (American Psychological Association n.d.). Further, the prevalence of anxiety disorders is two‐fold greater in women than men, and women often report higher illness burden and levels of disability than male peers with similar disorders (McLean et al. 2011). These findings suggest that males and females acquire mental health disorders at different rates, or possibly through different processes. Thus, identifying neural substrates that link ELS and GAD could potentially yield more targeted therapeutic interventions.

Prior research suggests ELS exerts a number of persistent physiological consequences on the body. These include changes in the function of immune cells, genetic transcription, and hormone release (Syed and Nemeroff 2017). Of note, microglia play an important role in the developing brain (Bachiller et al. 2018; Cowan and Petri 2018; Wright‐Jin and Gutmann 2019; Bolton et al. 2022; Paolicelli et al. 2011). During development, microglia engage in synaptic pruning (Paolicelli et al. 2011), axonal pruning (Lim and Ruthazer 2021), and release trophic factors to protect neurons and promote synapse maturation (Ueno et al. 2013; Morizawa et al. 2022). Further, ELS can induce changes in microglia function resulting in consequences on neuronal development and behavior (Kato et al. 2012; Bolton et al. 2022). Stress, spanning a variety of models, can impact microglia density, especially in the prefrontal and hippocampal regions (Calcia et al. 2016). The presence of stress‐related hormones during development, such as glucocorticoids, epinephrine, and norepinephrine, disrupt microglia function leading to issues with neuronal excitability and circuit function (Burke et al. 2016; Frank et al. 2019). Further, ELS can dysregulate synaptic pruning by interfering with microglial function, thus increasing the activity of stress‐sensitive synapses (Bolton et al. 2021).

In the context of ELS and GAD, studies have identified microglia as key contributors to regulating behaviors associated with anxiety (Catale et al. 2020). Further, microglia's role in developing GAD following ELS may be dependent on the sex of the subject, the developmental window of the exposure, and the brain region investigated (Dziabis and Bilbo 2022; Paolicelli et al. 2022). As such, microglia's role in the development of anxiety is complex (Masuda et al. 2019).

In order to determine the role of microglia in the relationship between ELS and anxiety‐like behaviors, we utilized a rodent model of adolescent social isolation (aSI). Experimental models of rodent aSI systematically increase behavioral and physiological changes associated with GAD in humans (Chappell et al. 2013; Gilpin and Weiner 2017). One well‐established aSI model involves moving young male Long Evans rats from group housing (GH) to a socially isolated (single‐housed) condition from P28 to 70. This manipulation leads to the expression of numerous behavioral alterations including increased displays of anxiety‐like behaviors and increased ethanol consumption (Hall 1998; McCool and Chappell 2009). Though this manipulation has historically not created the same phenotypes in female subjects (Butler et al. 2014), we recently tested the hypothesis that an earlier social isolation window, from P21 to 42, might yield comparable anxiety‐like phenotypes in both sexes (Ortelli et al. 2023). Notably, this study found that presenting aSI earlier in development promoted anxiety‐like behaviors in female Long Evans rats. Despite this, there is a large gap in the literature surrounding the sex‐dependent effects of aSI on behavior and brain function (Shansky 2019). To begin to address this gap, the present study sought to leverage this aSI procedure designed for female rodents to determine whether ablating microglia during aSI could prevent some of the behavioral phenotypes observed in our previous study (Ortelli et al. 2023). The present study tested microglia ablation as a potential early intervention on the effects of social isolation by administering PLX3397, a drug known to ablate microglia, starting on the first day of the aSI window.

## 2 Materials and Methods

### 2.1 Animals

The present experiment utilized 33 female Long Evans rats from Inotiv (Indianapolis, IN). All experimental procedures were in accordance with the National Institutes of Health Guide for Care and Use of Laboratory Animals. The experimental procedures have been approved by Wake Forest University Institutional Animal Care and Use Committee.

### 2.2 Early Life Stress Model

Upon arrival on P21, the rats were randomly separated into GH (*n* = 16, 4 per cage) or aSI (*n* = 17 total, 1 per cage). GH animals were housed in a large cage with 3 other rats (33.0 × 58.7 cm; Nalgene, Rochester, NY), while aSI animals were housed alone in standard polypropylene cages (20.3 × 26.7 cm; Allentown Inc., Allentown, NJ). All animals were exposed to a 12‐h/12‐h light/dark cycle, provided ad libitum access to food and water, and weighed weekly. The animals were kept in these housing conditions from P21 to P42–43 when tissue was collected.

### 2.3 PLX3397 Treatment

On P21, following housing assignment, animals were randomly assigned to a control diet or a PLX3397‐containing diet. PLX3397 inhibits colony‐stimulating factor 1 receptor (CSF1‐r). When provided over extended periods of time, PLX3397 ablates microglia and other myeloid lineage cells in the CNS (Elmore et al. 2014). AIN‐76A diet (AIN‐76A; Research Diets, New Brunswick, NJ) was utilized as a base for both control and PLX3397 diets. The composition of AIN‐76A is 20.3% protein, 65.9% carbohydrate, 5% fiber, and 5% fat. The PLX3397 diet included 600 mg/kg of PLX3397 (Pexidartinib) drug (MedChemExpress, South Brunswick Township, NJ) (Najafi et al. 2018).

### 2.4 Behavioral Testing

Each animal underwent a series of behavioral assays between P29 and P37. All animals were tested on the open field test (OFT), elevated plus maze (EPM), and successive alleys test (SAT) in that order. All behavioral tests were performed from 9:30 a.m. until approximately 12:30 p.m., with the animals being brought into the room 30 min prior to testing to acclimate. Each behavioral test was conducted over the course of two days, with half of the animals in each cohort being tested each day.

### 2.5 Open Field Test

On P29 and 30, the rats were assessed on an OFT. OFT is a well‐validated measure of locomotion (Turner and Burne 2014). Though locomotor activity is not a direct measure of anxiety‐like behavior, aSI evokes increased locomotor activity and is associated with behavioral phenotypes related to GAD and a vulnerability to develop substance use disorders (Chappell et al. 2013; Seibenhener and Wooten 2015). Examining distance traveled and movement time on the OFT can also give insight into more complex behaviors, such as lethargy. Notably, lethargic movement may indicate mood dysregulation in rodent models (Wang et al. 2018). Here, the rats were placed in an open chamber and their movement was recorded and analyzed for general locomotor activity (Prut and Belzung 2003). The activity box dimensions were 42 m x 42 cm x 30 cm and remained brightly lit for the duration of the 30‐min assay (Digiscan animal activity monitors, Omnitech, Columbus, OH, USA). Each rat was placed on this assay for 5 min. Each rat's behavior was recorded using Ethovision software (Noldus, Leesburg, VA).

### 2.6 Elevated Plus Maze

On P31 and 32, the rats were tested on a standard EPM (Med Associates Inc., Fairfax, VT). The EPM has been commonly used to test rodent's anxiety‐like behaviors (Fernandes et al. 1999). This apparatus contains 2 closed arms and 2 open arms (42 inch x 42 inch x 42.25 inch), which intersect to form a plus (+) shape. Here, rats were placed in the center of the maze, and their activity was recorded for 5 min. Increased time in the closed arms was operationally defined as increased anxiety‐like behavior. Each rat's behavior was recorded using Ethovision software (Noldus, Leesburg, VA).  As the black and white pattern on Long Evans rats can sometimes cause errors with Ethovision's placement on darker assays, each recording was looked over manually to ensure accuracy.

### 2.7 Successive Alleys Test

On P36 and 37, the SAT was performed (Deacon 2013). This assay is similar to the EPM as it is elevated off the floor, but instead of a cross‐like design, it has four distinct but continuous sections, each of which is 45 cm long. The first segment is colored dark gray and known as the “enclosed” zone (9.0 cm width, 29.0 cm wall height). The next segment is also colored dark gray with a much lower wall height (9.0 cm width, 2.5 cm wall height), which is followed by a light gray segment (6.7 cm width, 0.5 cm wall height) and finally a white (3.5 cm width, 0.3 cm wall height), which provides maximal exposure. Anxiety‐like behavior was measured by examining time spent in each zone, with more time in the lighter segments indicating less anxiety‐like behavior. Each rat was placed on this assay for 5 min. Each rat's behavior was recorded using Ethovision software and confirmed using manual scoring (Noldus, Leesburg, VA).

### 2.8 Tissue Collection

On P42 and 43, transcranial perfusions were performed with ice‐cold Del‐Bucco's phosphate buffered saline (dPBS) + 10 mg/L heparin at a flow rate of 8mLs/min for 3 min. Following this, brains were extracted and left in 10% formalin for 48 h before being transferred and stored in cryoprotectant (75% 0.05 M PBS, 10% glycerol, and 15% ethylene glycol). The brains were then sliced at 40 µm, utilizing a microtome from Boyle Instruments, before being stored again in a 30% sucrose cryoprotectant solution.

### 2.9 Histological Procedure

Tissue slices containing regions of interest including the prefrontal cortex (PFC), basolateral amygdala (BLA), and ventral Hippocampus (vHIP) were sorted and stored in 1 x PBS at 4°C. Slices were then washed for 3 × 5 min in 1 x PBS while rocking at room temperature. Following washes, tissue was transferred to a blocking solution (5% normal goat serum in PBS + 0.01% tween 80) for 1 h at room temperature while rocking. After blocking, slices were incubated in anti‐rabbit IBA‐1 (1:500; FUJIFILM Wako Pure Chemical Corporation; Catalogue #019‐19741), suspended in the blocking buffer, and kept at 4°C overnight while rocking. The following morning, the slices were washed using 1 x PBS 3 × 5 min, before secondary incubation. Slices were incubated in goat anti‐rabbit Alexa Fluor 488 diluted in the blocking buffer stated above (1:1000; Thermo Fisher Scientific; A32727) at room temperature for 2 h while covered and rocking. Following an additional round of 1 x PBS washes (3 × 5 min), the slices were exposed to DAPI (1:1000; Thermo Fisher Scientific; D1306) for 3 min before getting a final PBS wash. Lastly, these slices were mounted and cover‐slipped using aqua‐fluoromount. They were then imaged using a Keyence digital microscope (Keyence BZ‐X700; Itasca, IL) on 2x magnification. Additional representative images were taken using a 20x magnification.

### 2.10 Area Fraction Analysis

PFC, BLA, and vHIP slices were used to determine microglia population density. To determine microglia population density, we utilized the built‐in area fraction analysis in fiji (Green et al. 2022). Area fraction analysis calculates the percent area coverage of a given marker such as IBA‐1^+^ (Shoham et al. 2019; Green et al. 2022; Torres‐Rodriguez et al. 2023). Area fraction analysis was performed by three independent blinded researchers. Each researcher prepared slice images by thresholding to ensure no background noise would interfere with the appropriate IBA‐1^+^ signal. Once thresholded regions of interest were outlined (Paxinos and Watson 2014), the percent of that region that was occupied by microglia (IBA‐1^+^ cell) was recorded (Pait et al. 2024). Three slices were stained in each region for each animal. Some slices were excluded from the analysis due to artifacts and issues with signal‐to‐noise ratio. After performing the area fraction analysis, each reviewer averaged the results of slices within each respective region and rat. Scores for each animal and region were averaged for all 3 reviewers to give a final microglial percent area coverage. Due to variance in reviewer reports, area fraction analysis reduces confounding factors like rating error and variance in region size between slices when compared to traditional cell counting approaches.

### 2.11 Statistical Analysis

Statistical analyses were conducted using GraphPad Prism 10.1. For all analyses, two‐way ANOVAs were performed to identify the main effects of treatment and housing as well as treatment x housing interactions. Significant differences were identified at the cut‐off of *p* < 0.05. If a main effect or interaction was observed, Tukey's post hoc analyses were performed to determine further group differences.

## 3 Results

### 3.1 PLX3397 Reduced Microglia's Area Coverage in the PFC, BLA, and vHIP

**FIGURE 1:**
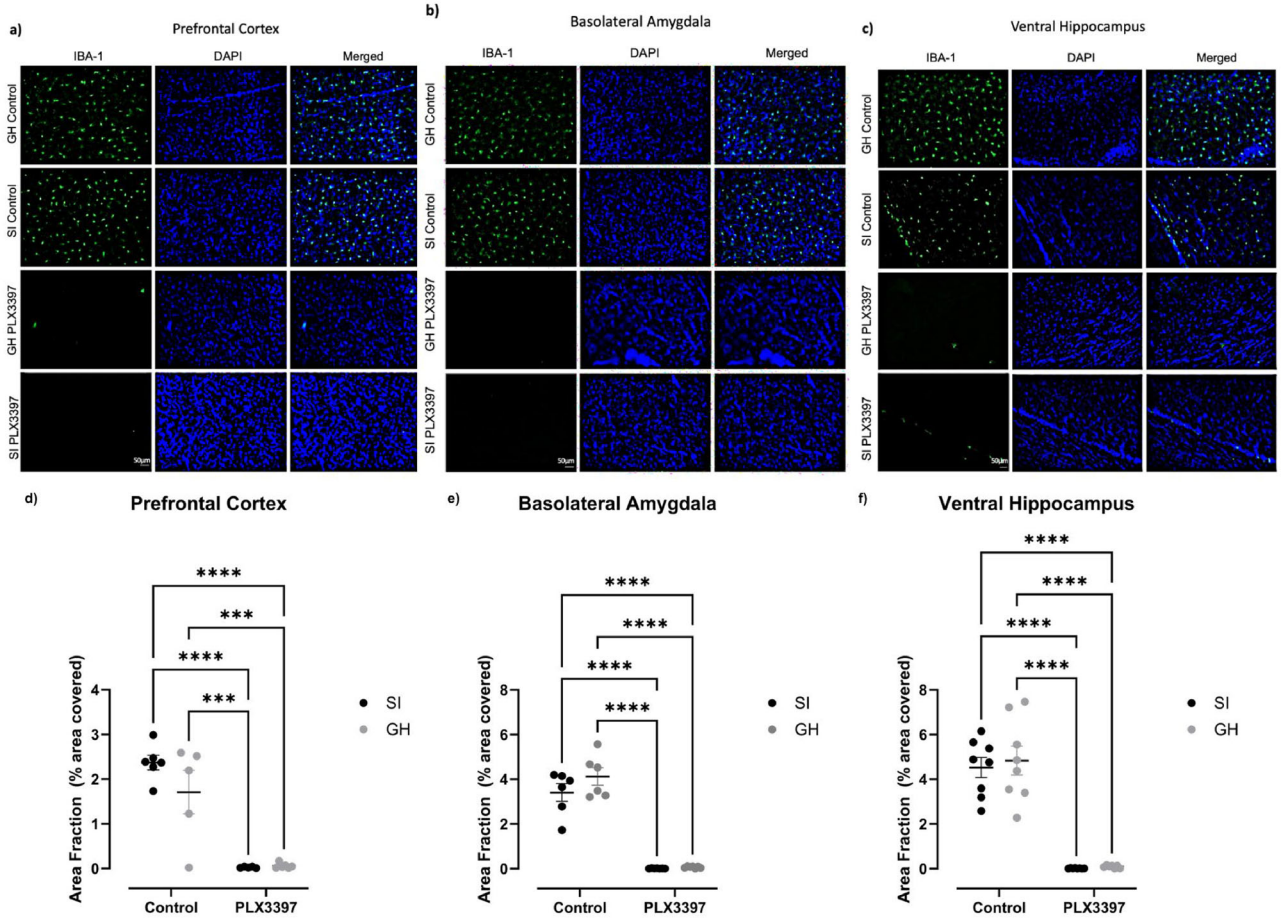
Three weeks of PLX3397 treatment ablates microglia populations in the PFC, BLA, and vHIP. a‐c) Representative images of IBA‐1^+^ microglia in green, nuclear marker DAPI in blue, and merged image for the PFC, BLA, and vHIP. d‐f) PLX3397 treatment reduced the number of IBA‐1^+^ cells in the PFC, BLA, and vHIP. *=p<.05, **=p<.01, ***=p<.001, ****=p<.0001. Scale bars in SI PLX3397 IBA‐1 pictures indicate 50µm.

To confirm that PLX3397 treatment ablated microglia, we examined the expression of the microglia/macrophage‐specific protein IBA‐1 in control and PLX3397 subjects in three brain regions known to contribute the maladaptive phenotypes promoted by aSI: the PFC, BLA, and vHIP (Kessler et al. 2010). Treatment with PLX3397 was effective at ablating microglia across all regions investigated as illustrated by representative staining images for the PFC (Figure 1a), BLA (Figure 1b), and vHIP (Figure 1c). In the PFC, two‐way ANOVA analysis revealed a main effect of treatment (Figure 1d; F(1,18) = 70.19, *p* < 0.0001), no effect of housing (Figure 1d; F(1,18) = 1.724, *p* = 0.2057), and no interaction between factors (Figure 1d; F(1,18) = 2.106, *p* = 0.1639). Similar findings were observed in the BLA, where a main effect of treatment was observed (Figure 1e; F(1,22) = 214.3, *p* < 0.0001) but no effect of housing (Figure 1e; F(1,22) = 2.303, *p* = 0.1434) and no interaction between factors (Figure 1e; F(1,18) = 1.684, *p* = 0.2079). In the vHIP, a main effect of treatment was also observed (Figure 1f; F(1,27) = 128.1, *p* < 0.0001). There was no main effect of housing (Figure 1f; F(1,27) = 0.2222, *p* = 0.6411) and no interaction between factors (Figure 1f; F(1,27) = 0.0837, *p* = 0.7745). In all observed regions, PLX3397 ablated microglia to a similar extent in both the aSI and GH subjects.

### 3.2 Effects of aSI and PLX3397 Treatment on Behavior in the OFT

**FIGURE 2:**
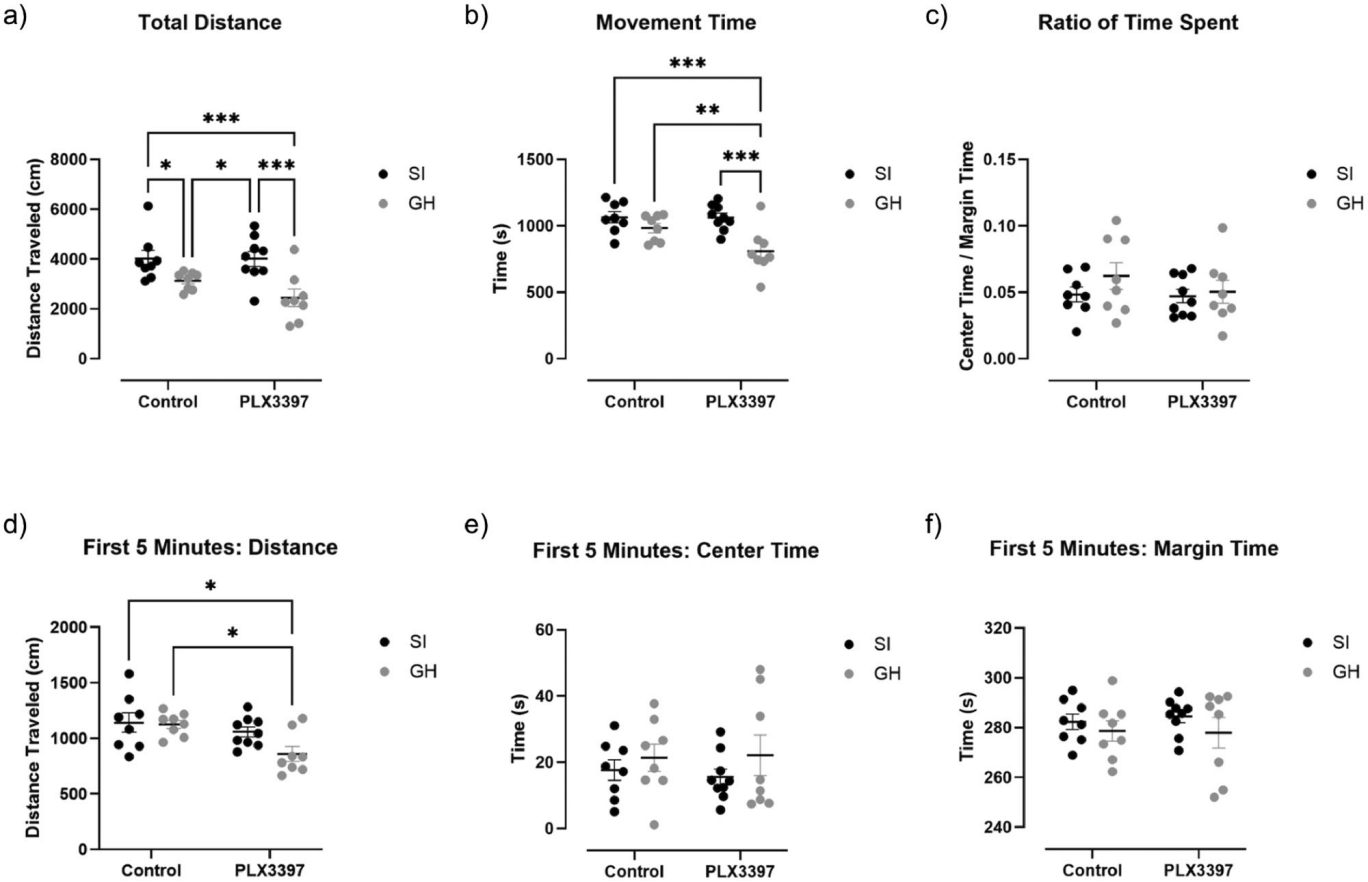
aSI increases total distance traveled and movement time on the open field test.

When assessing anxiety‐like behaviors on the OFT, a two‐way ANOVA revealed a main effect of housing on total distance traveled (Figure 2a, F(1,29) = 17.62, *p* = 0.0002). However, no main effect of PLX3397 treatment (Figure 2a, F(1,29) = 1.418, *p* = 0.2434), nor an interaction (Figure 2a, F(1,29) = 1.361, *p* = 0.2528) were observed. Post hoc testing revealed that aSI control subjects traveled larger distances than the GH control group (*p* = 0.0433) and SI PLX3397 rats traveled larger distances than GH PLX+ subjects (*p* = 0.0006). As such, PLX3397 treatment had no effect on this expected aSI phenotype. For total movement time, there was a main effect of housing (Figure 2b, F(1,29) = 14.89, *p* = 0.0006), a strong trend towards a main effect of PLX3397 treatment (Figure 2b, F(1,29) = 4.000, *p* = 0.0549), and an interaction between factors (Figure 2b, F(1,29) = 3.946, *p* = 0.0565). Surprisingly, post hoc analysis revealed that only aSI rats increased movement time in the PLX3397 subjects (*p* = 0.0002). When examining the ratio of time spent in the center as opposed to the margin, there was no significant effect of PLX3397 treatment (Figure 2c, F(1,29) = 0.7672, *p* = 0.3883), housing (Figure 2c, F(1,29) = 1.301, *p* = 0.2633), or interaction (Figure 2c, F(1,29) = 0.4895, *p* = 0.4897). When looking at the total distance traveled within the first 5 min of testing, there was a significant effect of PLX3397 treatment (Figure 2d, F(1,29) = 8.183, *p* = 0.0078), but not housing (Figure 2d, F(1,29) = 3.087, *p* = 0.0895), or interaction (Figure 2d, F(1,29) = 2.263, *p* = 0.1433). Post hoc testing revealed that GH PLX3397 animals moved less than GH controls (*p* = 0.0241) or SI controls (*p* = 0.0158). When looking at the center time for only the first 5‐min interval, there was no significant effect of PLX3397 treatment (Figure 2e, F(1,29) = 0.0267, *p* = 0.8713), housing (Figure 2e, F(1,29) = 1.563, *p* = 0.2212), or interaction (Figure 2e, F(1,29) = 0.1225, *p* = 0.7289). This remains true for margin time during the first 5 min, as there is no significant effect for PLX3397 treatment (Figure 2f, F(1,29) = 0.0266, *p* = 0.8715), housing (Figure 2f, F(1,29) = 1.563, *p* = 0.2213), or interaction (Figure 2f, F(1,29) = 0.1227, *p* = 0.7287).

In addition, we looked at behavior on the OFT in 5‐min bins. Here, we visually depicted time spent in the center (Figure S1a), time spent on the margin (Figure S1b), and the ratio of center versus margin time (Figure S1c) throughout the 30‐min test. Further, when looking at correlations between microglia density in the PFC and behavior, there was a significant correlation among control aSI animals when examining OFT total distance traveled (Figure S2a, *r* = 0.8700, *p* = 0.02). Similarly, there was a near‐significant correlation between microglia density in the vHIP and total distance traveled (Figure S2f, *r* = −0.7332, *p* = 0.06), as well as a significant correlation with total movement time (Figure S2f, *r* = −0.8767, *p* = 0.01) among PLX3397 treated GH animals.

### 3.3 Effects of aSI and PLX3397 Treatment on the EPM

**FIGURE 3:**
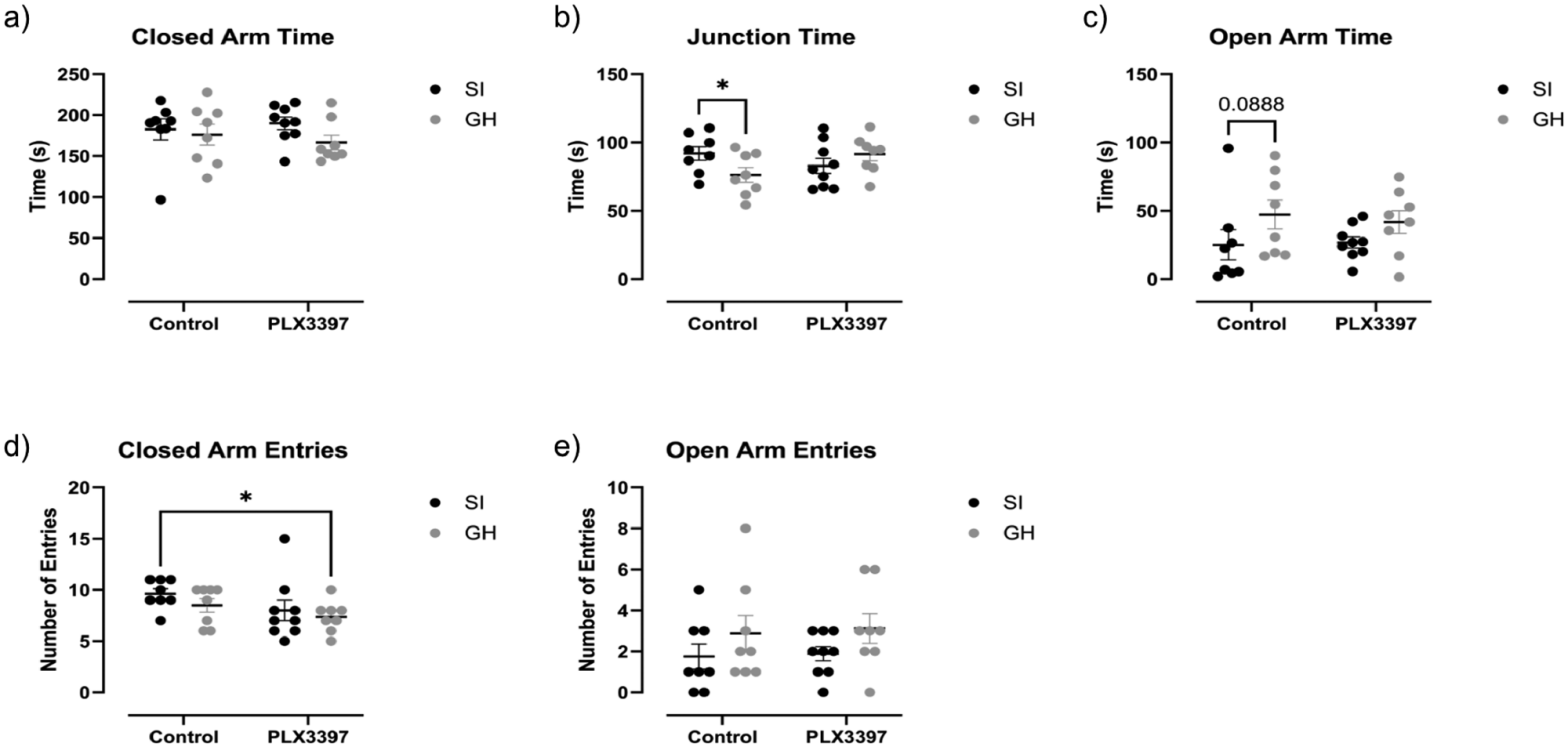
Housing and PLX3397 treatment do not alter behavior on the EPM. a) There was no significant effect of PLX3397 treatment, housing, or interaction when examining closed arm time. b) There was a signficant interaction effect between PLX3397 treatment and housing when examining time spent in the junction (p=0.0276) as aSI controls displayed significantly more junction time than GH controls. c) For open arm time, there was a significant effect of housing (p=0.0433), but not PLX3397 treatment or interaction. d,e) There was no significant effect of PLX3397 treatment or housing when considering the number of closed arm entries and open arm entries *=p<.05.

On the EPM, a two‐way ANOVA analyzing closed arm time showed no significant effect of PLX3397 treatment (Figure 3a, F(1,29) = 0.0099, *p* = 0.9214), housing (Figure 3a, F(1,29) = 1.958, *p* = 0.1723), or interaction (Figure 3a, F(1,29) = 0.6340, *p* = 0.4324). When looking at time spent in the junction, there was no significant effect of PLX3397 treatment (Figure 3b, F(1,29) = 0.3221, *p* = 0.5747) or housing (Figure 3a, F(1,29) = 0.4735, *p* = 0.4969), but there was a significant interaction effect (Figure 3a, F(1,29) = 5.378, *p* = 0.0276). Post hoc analysis revealed a significant difference between control GH and aSI rats (*p* = 0.0449). Similarly, when examining open arm time, there was a significant effect of housing (Figure 3c, F(1,29) = 4.468, *p* = 0.0433), but not PLX3397 treatment (Figure 3c, F(1,29) = 0.0453, *p* = 0.8330) or interaction (Figure 3c, F(1,29) = 0.1700, *p* = 0.6832). When examining the number of closed arm entries, a measure of general locomotor activity, there was no effect of housing (Figure 3d, F(1,29) = 1.453, *p* = 0.2378), or interaction between factors (Figure 3d, F(1,29) = 0.1186, *p* = 0.7330). However, a trend towards an effect of PLX3397 treatment (Figure 3d, F(1,29) = 3.588, *p* = 0.0682) was observed. Lastly, a two‐way ANOVA revealed a trending decrease in open arm entries in SI subjects (Figure 3e, F(1,29) = 3.256, *p* = 0.0816), no effect of PLX3397 treatment (Figure 3e, F(1,29) = 0.0883, *p* = 0.7684), and no interaction between factors (Figure 3e, F(1,29) = 0.072, *p* = 0.9329). Ultimately, PLX3397 treatment did not significantly change the behavior of the EPM.

In addition, there was a significant correlation between microglia density in the PFC and duration in the enclosed arms among PLX3397‐treated GH animals (Figure S2b, *r* = 0.8665, *p* = 0.03). Also, there was a significant correlation between microglia density in the BLA and open‐arm entries among PLX3397‐treated aSI animals (Figure S2c, *r* = −0.7736, *p* = 0.04). Similarly, there was a significant correlation between microglia density in the BLA and closed‐arm entries among control GH animals (Figure S2d, *r* = −0.8930, *p* = 0.02). Lastly, there was a significant correlation when comparing microglia density in the vHIP with open arm entries among PLX3397 treated aSI animals (Figure S2e, *r* = −0.7410, *p* = 0.04)

### 3.4 Effects of aSI and PLX3397 Treatment on Behavior in the SAT

**FIGURE 4:**
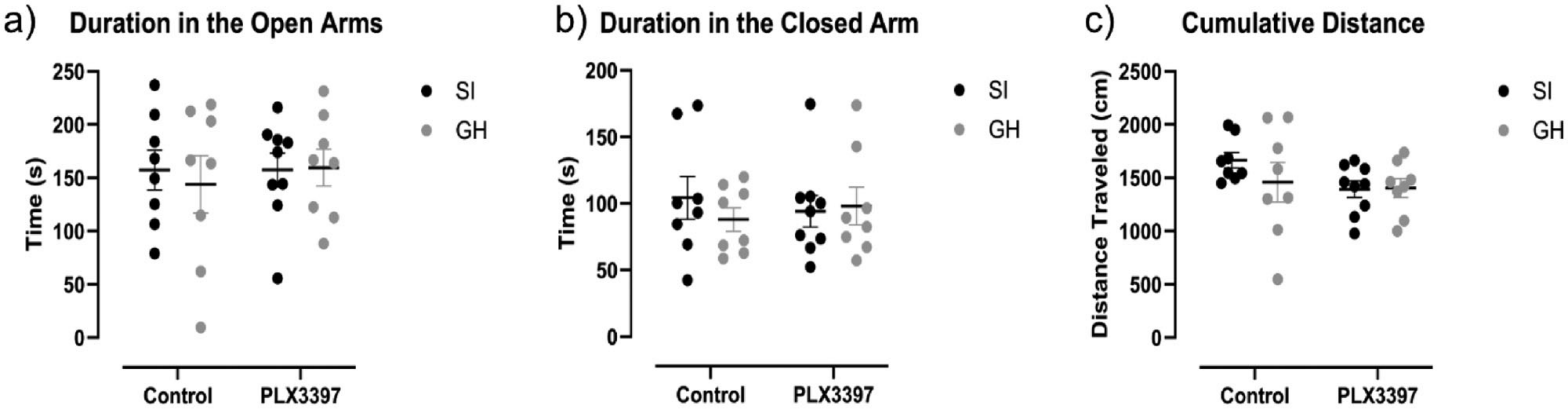
Housing and PLX3397 treatment do not alter behavior on the SAT. a‐c) There was no effect of housing or PLX3397 treatment on time spent in the open arms, closed arm, or distance traveled.

After completing the EPM, rats were tested on the SAT. A two‐way ANOVA revealed no effect of housing (Figure 4a, F(1,29) = 0.7334, *p* = 0.3985), PLX3397 treatment (Figure 4a, F(1,29) = 2.049, *p* = 0.1630), or an interaction between factors (Figure 4a, F(1,29) = 0.9134, *p* = 0.3471) on cumulative distance traveled. When considering cumulative duration in the open arms, there were also no effects of housing (Figure 4b, F(1,29) = 0.0820, *p* = 0.7767), PLX3397 treatment (Figure 4b, F(1,29) = 0.1573, *p* = 0.6945), or an interaction between factors (Figure 4b, F(1,29) = 0.1522, *p* = 0.6993). Finally, a two‐way ANOVA also demonstrated no effect of housing (Figure 4c, F(1,29) = 0.2285, *p* = 0.6363), PLX3397 treatment (Figure 4c, F(1,29) = 2.272e‐005, *p* = 0.9962), or an interaction (Figure 4c, F(1,29) = 0.6116, *p* = 0.4405) on duration in the closed arm.

Further, there was a significant correlation when comparing microglia density in the vHIP with duration in the open areas (Figure S2e, *r* = −0.7664, *p* = 0.03) and distance traveled (Figure S2e, *r* = −0.7786, *p* = 0.02) among PLX3397 treated aSI animals.

## 4 Discussion

Despite extensive research, the link between ELS and an increased risk of developing anxiety disorders is not fully understood, particularly in women (Kessler et al. 2010; Lähdepuro et al. 2019). This gender gap can be attributed, in part, to a lack of preclinical research that has included female subjects in their studies (McCool and Chappell 2009; Oyola and Handa 2017; An et al. 2022). We employed a modified aSI that promotes relevant anxiety‐related behavioral phenotypes in female rats (Ortelli et al. 2023) to examine whether microglia contribute to some of these behaviors. Previous studies have demonstrated that ELS can disrupt microglia phagocytic activity and can prime microglia to be more reactive later in life (Burke et al. 2016; Paolicelli and Ferretti 2017). These microglial changes have been shown to be persistent throughout life and to have an impact on behavior.

This study focused on microglia's involvement in the effects of aSI on anxiety‐like behaviors in female rats. Treatment with PLX3397, a CSF‐1r inhibitor, significantly reduced microglia levels in all observed areas, including multiple regions associated with anxiety, namely the PFC, BLA, and vHIP (Figure 1a–f). After confirming that the PLX3397 treatment successfully reduced microglia density, each animal's behavior was analyzed. We found that relative to GH rats, aSI significantly increased locomotor activity as assessed by total distance traveled (Figure 2a) and movement time. These data replicate previous findings with this ELS model (Ortelli et al. 2023). Further, increased locomotor activity has been linked to anxiety‐like behaviors through manipulations involving anxiolytic drugs (Sainati and Lorens 1983; Djeridane et al. 2005). Importantly, PLX3397 treatment did not significantly block these aSI‐induced increases in locomotor activity.

On P31 and 32, subjects were assessed on the EPM. Although not statistically significant, open‐arm entries were relatively lower in aSI animals independent of PLX3397 treatment. It is important to note that aSI did not significantly increase this measure in females in our other recent studies (Ortelli et al. 2023; Pitcairn et al. 2024) and there is evidence that this assay may not engender male‐like anxiety‐like phenotypes in females (Fernandes et al. 1999; Rivera‐Irizarry et al. 2023). Regardless, PLX3397 treatment did not alter anxiety‐like behaviors.

Finally, neither housing nor PLX3397 treatment drove behavioral differences on the SAT when examining total distance traveled or cumulative duration in the open and enclosed areas. Importantly, to our knowledge, very few studies have used the SAT to assess anxiety‐like behavior in female rats, and none have employed this assay to assess SI‐associated changes in anxiety‐like behavior (Bach et al. 2021; Pitcairn et al. 2024). It is possible that variables such as the nutritional composition of the diet, and the specific stress procedure utilized in this study have contributed to the behavioral differences observed on the SAT. Regardless, PLX3397 treatment had no significant effect on behavior.

Overall, findings from the present study suggest that microglial ablation during aSI had a minimal effect on anxiety‐like behaviors. These findings add to existing literature investigating the effects of ELS on microglial function and behavioral outcomes (Catale et al. 2019; Catale et al. 2020). There are a number of potential interpretations of these behavioral results. First, microglia do not regulate the effects of aSI on anxiety‐like behaviors. PLX3397 treatment and subsequent microglia ablation did not substantially alter behavior as assessed by the OFT, EPM, and SAT. Second, PLX3397 does have off‐target effects (Claeys et al. 2023). PLX3397's interaction with other macrophage types and peripheral immune cells potentially altered the behavioral profiles observed (Claeys et al. 2023). Third, microglia phenotypes may be under‐classified. Microglia are often labeled using the binary terms: “M1/inflammatory” or “M2/anti‐inflammatory” (Guo et al. 2022). However, microglia serve a vast number of functions around the central nervous system, and thus, their classification should match the complexity of their states and functions (Woodburn et al. 2021; Paolicelli et al. 2022). To exclude microglia's potential involvement in mediating the relationship between ELS and anxiety‐like behaviors, further studies should fully consider the diverse array of functions and phenotypes associated with microglia (Leyh et al. 2021; Wang et al. 2023). Therefore, it will be important to develop methods to selectively target microglia subtypes in future studies.

Specifically, chow‐based PLX3397 has been shown to deplete microglia activity within seven days of administration (Elmore et al. 2014). As PLX3397 administration began on P21, microglia were depleted by the time testing began on P29. Despite this, microglia may not have been depleted for the entirety of the aSI paradigm. An earlier microglia depletion spanning the entirety of the aSI window could have altered anxiety‐like phenotypes in a way not captured by the present experiment. Further, reviews noted that many previous studies have observed elevated IBA‐1 levels in response to stress, though this is not always the case. Though we did not observe significant differences in microglia levels in response to stress as expected, this is likely a result of previous studies not examining ELS through aSI in female rats specifically. Notably, a previous study observed that ELS through social prolonged stress significantly increased microglia density in male rats, but this effect was not seen in females (Torres‐Rodriguez et al. 2023).

Potential follow‐up experiments could further assess how a PLX3397 treatment impacts anxiety‐like behaviors using other behavioral assays and ELS models. ELS models of interest include single prolonged stress (SPS) (Lisieski et al. 2018), or social defeat stress (SDS) (Iñiguez et al. 2014), while other behavioral assays of note include light/dark box and forced swim test (Castagné et al. 2011). Further, recent work from our lab has investigated the effects of aSI on voluntary ethanol self‐administration (Ortelli et al. 2023). Identifying whether PLX3397 can blunt the effects of aSI on ethanol consumption represents another important future direction. Follow‐up studies can examine the impact of this aSI model on the nucleus accumbens, a region previously associated with microglial changes in response to ELS (Kopec et al. 2018).

In addition, in order to better understand and test how aSI impacts female rats, future studies should focus on characterizing aSI‐related phenotypes specifically in female subjects. As PLX3397 did not significantly alter anxiety‐like traits associated with aSI in the present experiment, it should be noted that aSI only had a minimal effect on these traits. This is consistent with previous studies that have observed limited changes to anxiety‐like behaviors in response to microglia ablations, though in control animals (Basilico et al. 2022). Finding additional testable behaviors, both immediately after a stress manipulation and later in adulthood, that are more sensitive to characterizing aSI effects in females specifically may allow for a more comprehensive analysis of how microglia depletion impacts behavior in female rats.

## 5 Conclusion

The present study suggests that PLX3397 is not sufficient to significantly alter phenotypes associated with aSI and anxiety‐like behaviors in female rats. Though our findings do not implicate microglia in the maladaptive behaviors associated with aSI, more studies will be needed to fully determine the relationship between various forms of ELS and microglial function.

## Author Contributions

**Matthew A. Blumberg**: conceptualization, investigation, writing–original draft, methodology, writing–review and editing. **Ava Shipman**: investigation. **Lidia Olyha**: investigation. **Stephen C. Gironda**: conceptualization, writing–original draft, investigation, methodology, writing–review and editing. **Jeffrey L. Weiner**: conceptualization, funding acquisition, writing–review and editing.

### Peer Review

The peer review history for this article is available at https://publons.com/publon/10.1002/brb3.70369.

## Supporting information



Figure S1: OFT behavior throughout 30 min test.
Figure S2: Significant correlations between microglia density and behavior.

## Data Availability

The data that support the findings of this study are available from the corresponding author upon reasonable request.
