# Supplementary material for: Is the Relationship Between Adolescent Social Isolation and Anxiety‐Like Behaviors Altered by Microglia Ablation in Female Long Evans Rats?
Source: Brain Behav. 2025 Mar 9;15(3):e70369. doi: 10.1002/brb3.70369 (PMC11891277; doi:10.1002/brb3.70369)
Supplement: Supplementary file 1 — Figure S1: OFT behavior throughout 30 min test. Figure S2: Significant correlations between microglia density and behavior. [file BRB3-15-e70369-s001.docx]

**Figure S1: OFT behavior throughout 30 minute test**


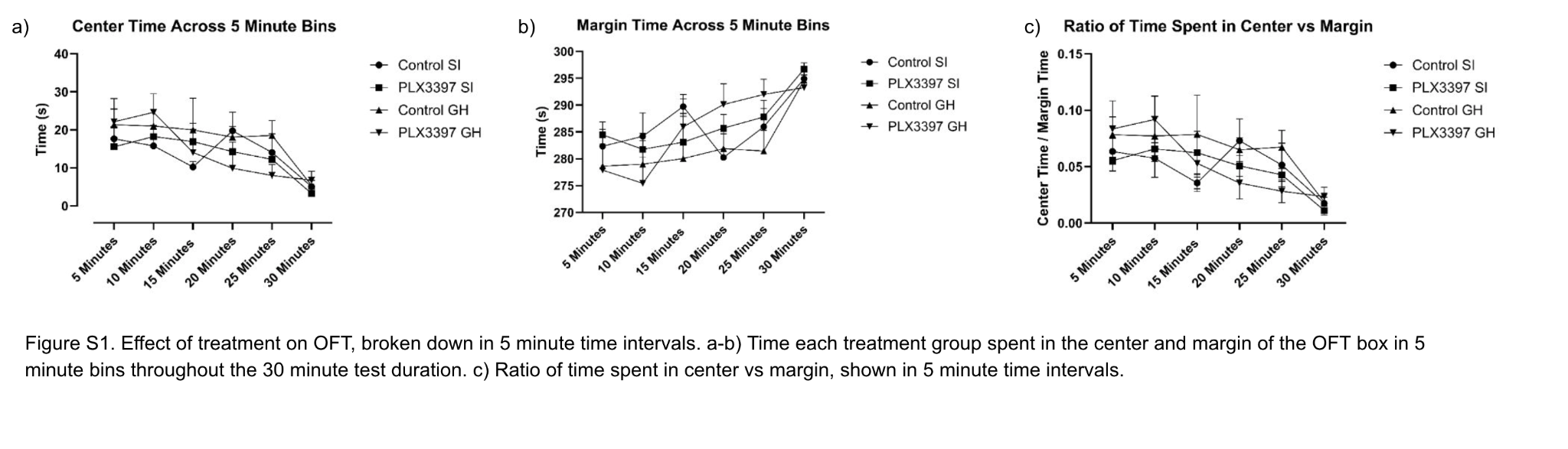


**Figure S2: Significant correlations between microglia density and behavior**

**
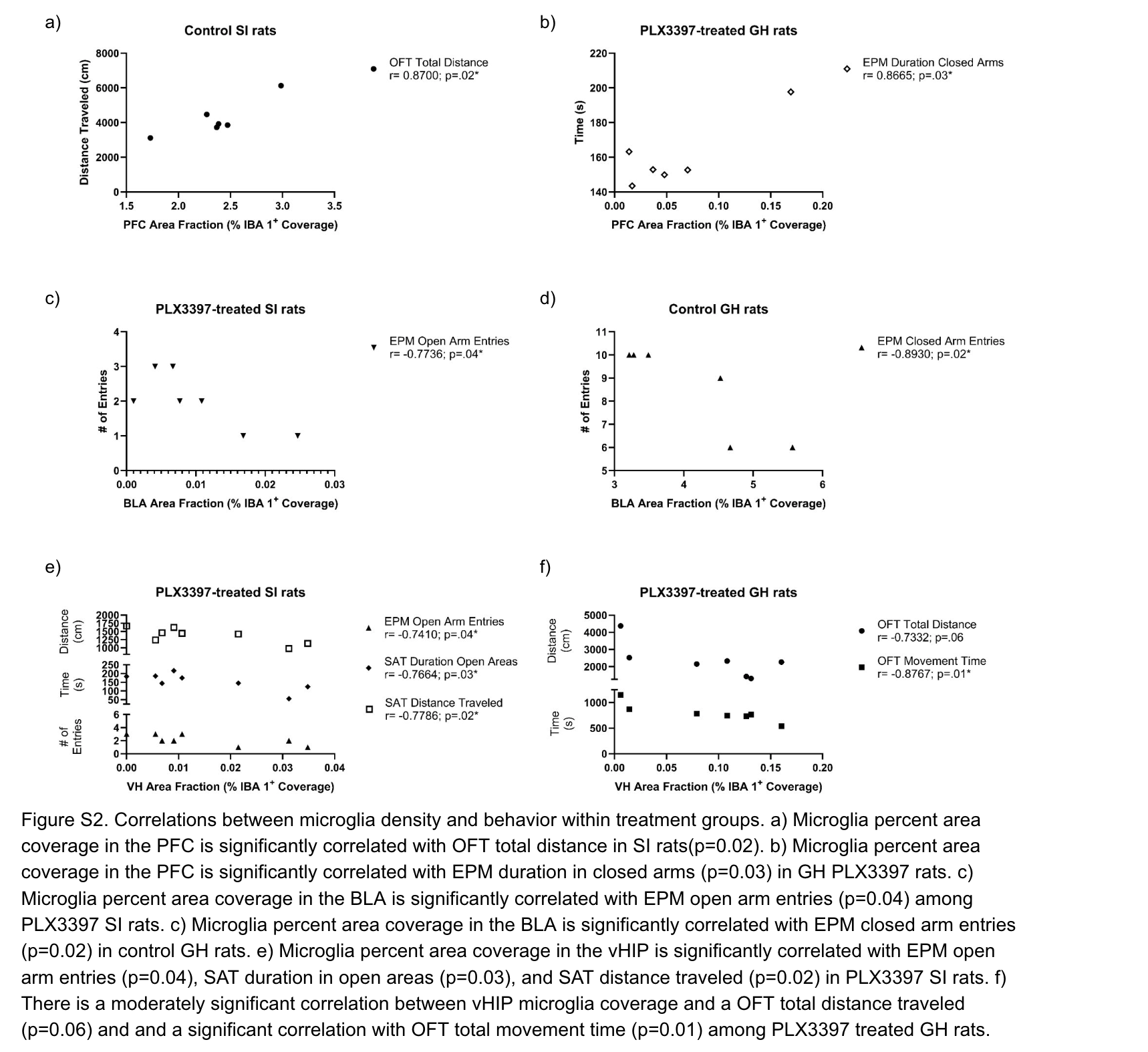
**
